# Synergy of ruthenium metallo-intercalator, [Ru(dppz)_2_(PIP)]^2+^, with PARP inhibitor Olaparib in non-small cell lung cancer cells

**DOI:** 10.1038/s41598-023-28454-x

**Published:** 2023-01-26

**Authors:** Nur Aininie Yusoh, Suet Lin Chia, Norazalina Saad, Haslina Ahmad, Martin R. Gill

**Affiliations:** 1 UPM-MAKNA Cancer Research Laboratory, Institute of Bioscience, Universiti Putra Malaysia, 43400 Serdang, Selangor Malaysia; 2 Department of Microbiology, Faculty of Biotechnology and Biomolecular Science, Universiti Putra Malaysia, 43400 Serdang, Selangor Malaysia; 3 Department of Chemistry, Faculty of Science, Universiti Putra Malaysia, 43400 Serdang, Selangor Malaysia; 4 Department of Chemistry, Faculty of Science and Engineering, Swansea University, Swansea, UK

**Keywords:** Mechanism of action, Metals, Pharmacology, Pharmacology, Toxicology, Chemical biology, Lung cancer

## Abstract

Poly(ADP-ribose) polymerase (PARP) are critical DNA repair enzymes that are activated as part of the DNA damage response (DDR). Although inhibitors of PARP (PARPi) have emerged as small molecule drugs and have shown promising therapeutic effects, PARPi used as single agents are clinically limited to patients with mutations in germline breast cancer susceptibility gene (BRCA). Thus, novel PARPi combination strategies may expand their usage and combat drug resistance. In recent years, ruthenium polypyridyl complexes (RPCs) have emerged as promising anti-cancer candidates due to their attractive DNA binding properties and distinct mechanisms of action. Previously, we reported the rational combination of the RPC DNA replication inhibitor [Ru(dppz)_2_(PIP)]^2+^ (dppz = dipyrido[3,2-*a*:2′,3′-*c*]phenazine, PIP = 2-(phenyl)-imidazo[4,5-*f*][1,10]phenanthroline), “Ru-PIP”, with the PARPi Olaparib in breast cancer cells. Here, we expand upon this work and examine the combination of Ru-PIP with Olaparib for synergy in lung cancer cells, including in 3D lung cancer spheroids, to further elucidate mechanisms of synergy and additionally assess toxicity in a zebrafish embryo model. Compared to single agents alone, Ru-PIP and Olaparib synergy was observed in both A549 and H1975 lung cancer cell lines with mild impact on normal lung fibroblast MRC5 cells. Employing the A549 cell line, synergy was confirmed by loss in clonogenic potential and reduced migration properties. Mechanistic studies indicated that synergy is accompanied by increased double-strand break (DSB) DNA damage and reactive oxygen species (ROS) levels which subsequently lead to cell death via apoptosis. Moreover, the identified combination was successfully able to inhibit the growth of A549 lung cancer spheroids and acute zebrafish embryos toxicity studies revealed that this combination showed reduced toxicity compared to single-agent Ru-PIP.

## Introduction

Lung cancer is one of the most prevalent type of diseases and remains the leading cause of cancer-related death^1,2^. Among the classified lung cancers, non-small cell lung cancer (NSCLC) accounts for 85% of the total lung cancer cases reported, with the remaining 15% of total cases are small cell lung cancer (SCLC)^3^. Current treatments for NSCLC patients include surgical resection alongside adjuvant chemotherapy for early-stage disease, and chemoradiation for advanced disease. Cisplatin, or cis-diamminedichloroplatinum(II), is still to date the preferred drug for adjuvant chemotherapy in the treatment of NSCLC patients. Cisplatin induces inter- and intra-strand platinum–DNA adducts resulting in cytotoxic DNA double-strand breaks (DSBs)^4,5^. However, despite effective in many cases, cisplatin is clinically limited due to high general toxicity with severe adverse effects have been reported, including nephrotoxicity, ototoxicity and neurotoxicity^6^. Moreover, the emergence of resistance during prolonged cisplatin treatment remains one of the major challenges in the clinical use of cisplatin^7^. As such, the development of new anti-cancer metallocompounds to improve upon the limitations of cisplatin are required, and the rational combination with DNA damage repair inhibitors has appeared as an attractive strategy for cancer combinatorial therapy^8,9^.

Poly(ADP-ribose) polymerase (PARP) are critical DNA repair enzymes that are involved in various cellular responses, particularly in DNA single-strand breaks (SSBs) repair to prevent the generation of cytotoxic DSBs^10^. Based on this background, exploiting DNA replication stress by targeting PARP enzymes in addition to selectively targeting cancer cells with deficiencies in homologous recombination (HR) signaling has shown therapeutic promise^11^. Since then, PARP inhibitors (PARPi) have been developed as anti-cancer agents and several are now FDA-approved^12–14^. Arguably, Olaparib (Lynparza®) is the most successful PARPi reported to date and has gained approval for treating ovarian and breast cancers harboring mutations in germline breast cancer BRCA1/2 genes^15^. However, BRCA-deficient cancers account for a relatively small subset of patients and, with very few exceptions, chemotherapy utilizing single-drug had not been successful in prolonging patient survival due to rapid emergence of drug resistance and thus, preventing long lasting clinical benefits of PARPi^16^. In recent studies, PARPi combinations with various DNA damaging chemotherapies, radiotherapies, immunotherapies and other targeted therapies have been examined in preclinical models and clinical trials, and as a result of drug synergisms, they have shown potential to tackle resistance to PARPi and treat cancers without BRCA mutation^17–19^.

**Figure 1:**
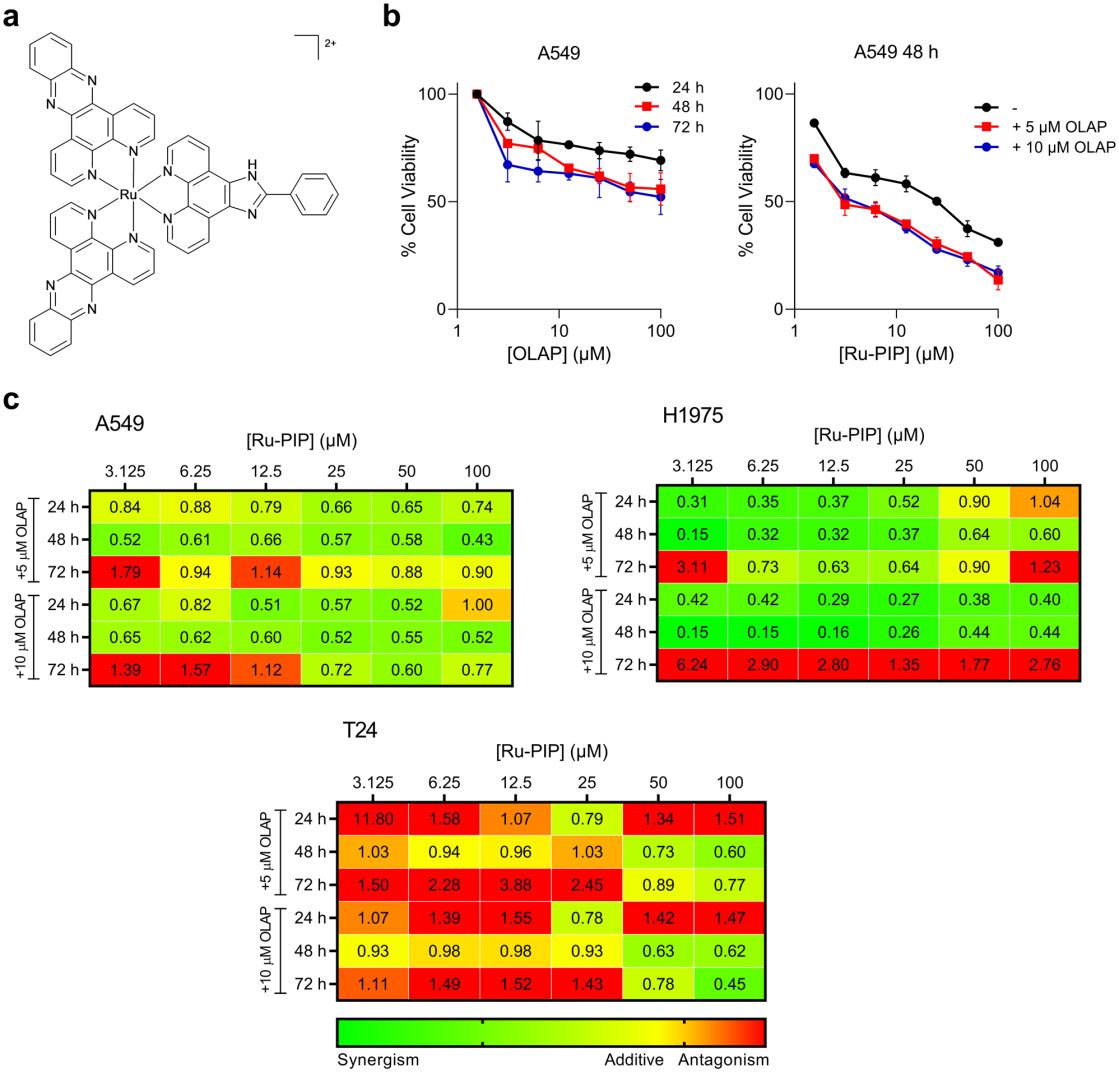
Ru-PIP synergizes with Olaparib in lung cancer cells. (**a**) Structure of Ru-PIP. (**b**) Left, cell viability of A549 cells upon treatment with Olaparib (OLAP). Right, cell viability of A549 cells upon treatment with Ru-PIP alone or in combination with Olaparib at 48 h. Mean ± SD of three independent experiments. (**c**) Combination indices (CIs) for Ru-PIP and Olaparib combinations in A549, H1975 and T24 cells for 24, 48 and 72 h treatment. CI values were calculated using CompuSyn software, and a heat map was generated as described within Methods section.

There has been an increasing interest in the anti-cancer activity of ruthenium compounds as alternatives to—or even replacements for—current platinum chemotherapy^20–23^. The majority of these ruthenium compounds are able to target DNA of highly proliferative cancer cells via the formation of ruthenium-DNA adducts, causing DNA damage to DNA and the inhibition of cellular proliferation in a manner analogous to cisplatin. However, over the last decade, substitutionally inert ruthenium polypyridyl complexes (RPCs) have emerged as promising anti-cancer agents where, in these cases, biomolecular binding is facilitated by the ligand(s) coordinated to the ruthenium metal center^24–26^. Most successful example of RPC includes the photosensitizer TLD1433 that has entered phase II clinical trials for bladder cancer patients. Moreover, recent clinical trials also showed that ruthenium complexes are able to re-sensitize platinum-resistant cancer to chemotherapy^27^. Previously, we have demonstrated that the RPC [Ru(dppz)_2_(PIP)]^2+^ (dppz = dipyrido[3,2-*a*:2′,3′-*c*]phenazine, PIP = 2-(phenyl)-imidazo[4,5-*f*][1,10]phenanthroline), also termed as Ru-PIP (Fig. 1a), inhibits DNA replication by intercalation of its organic ligand(s) between DNA base pairs, resulting in activation of DNA damage response (DDR) and G1 phase cell-cycle arrest^28^.

Considering that Ru-PIP treatment led to stalled DNA replication fork progression and PARP is activated upon DNA-strand break to prevent fork collapse, we hypothesized that this combination would result in sustained DNA damage leading to enhanced cytotoxic effect on cancer cells. Indeed, as a direct result of this mechanism of action, Ru-PIP hypersensitizes BRCA wild-type triple-negative breast cancer cells to PARPi Olaparib treatment^29^. This prompted us to investigate the possibility that PARPi may be employed to exacerbate the cytotoxic potential of RPCs in NSCLC cells. In this study, we evaluate the effects of Ru-PIP in combination with Olaparib in NSCLC – including a spheroid model—and explore the mechanistic basis on cell survival, migration, cell cycle arrest and induction of apoptosis. Given the importance of evaluating the safety profile of newly identified combination, an acute zebrafish embryos toxicity test was carried out to evaluate the toxic effects of this combination in comparison to as single agents. We demonstrate that the combination of Ru-PIP and Olaparib synergize in a representative NSCLC cell line to achieve profound anti-cancer effect in both 2D and 3D cell culture models with reduced toxicity effect on zebrafish embryos and thus, this may present a promising therapeutic strategy for NSCLC.

## Results

### Ru-PIP or Olaparib exhibit selective cytotoxicity towards lung cancer cells

To evaluate the effects of single agents Ru-PIP and Olaparib, three cancer cell lines were treated with concentration gradient of the stated compounds and the resultant inhibition on cells growth was observed using MTT assay. Normal MRC5 human lung fibroblasts were included for comparison. Ru-PIP showed greater potency in A549 lung cancer cells with 72 h half maximal inhibitory concentration (IC_50_) of 11.4 µM compared to H1975 lung cancer and T24 bladder cancer cells with 72 h IC_50_ of 59.4 µM and 15.8 µM, respectively (Table 1). In contrast to Ru-PIP, Olaparib showed minimal activity on these tested cancer cells with 72 h IC_50_ of > 71.7 µM (Figs. 1b, S1 and Table 1). Most importantly, Ru-PIP showed selective cytotoxicity towards cancer cells than in the normal MRC5 cells with high selectivity indices (SI) of > 1.6 upon 72 h treatments in A549, H1975 and T24 cells (Table S1). For comparison, the SI value obtained upon 72 h treatment with cisplatin in A549 cells in comparison to MRC5 cells is 2.5: substantially lower than that of Ru-PIP (72 h SI value of > 8.7) (Fig. S1 and Table S1). Moreover, in contrast to Ru-PIP, treatment with cisplatin in both H1975 and T24 cells did not result in any clear cancer selectivity activity with SI < 1. These findings indicate that Ru-PIP has noteworthy tumor-specific cytotoxic action with greater selectivity indices than cisplatin in the three cancer cell lines tested.

**Table 1:** Half maximal inhibitory concentration (IC_50_) values of Ru-PIP, Olaparib, Ru-PIP/Olaparib and cisplatin upon 24, 48 and 72 h treatment in A549, H1975, T24 and MCR5 cells. 100 μM is the maximum concentration tested. Mean ± SD of three independent experiments.

Compound	IC_50_ (µM)											
	A549			H1975			T24			MRC5		
	24 h	48 h	72 h	24 h	48 h	72 h	24 h	48 h	72 h	24 h	48 h	72 h
Ru-PIP	34.5 ± 10.5	19.4 ± 3.5	11.4 ± 0.3	> 100	95.1 ± 2.9	59.4 ± 11.6	45.1 ± 6.2	22.7 ± 2.8	15.8 ± 2.9	> 100	91.0 ± 7.9	> 100
Olaparib	> 100	> 100	> 100	> 100	> 100	71.7 ± 11.8	> 100	> 100	> 100	> 100	> 100	> 100
Ru-PIP + 5 µM Olaparib	12.8 ± 2.4	5.0 ± 0.1	7.0 ± 0.7	> 100	57.0 ± 6.0	31.5 ± 12.9	37.8 ± 9.1	15.9 ± 2.0	29.5 ± 10.7	61.1 ± 9.2	70.7 ± 5.4	70.6 ± 0.7
Ru-PIP + 10 µM Olaparib	9.8 ± 1.0	4.7 ± 0.3	7.4 ± 0.9	56.6 ± 1.6	24.9 ± 7.0	> 100	42.4 ± 4.3	12.8 ± 1.3	15.4 ± 3.8	62.1 ± 6.6	54.1 ± 0.9	66.0 ± 2.4
Cisplatin	6.6 ± 2.0	4.8 ± 0.5	2.9 ± 1.2	26.6 ± 9.9	20.0 ± 6.7	13.5 ± 5.7	59.6 ± 7.0	20.8 ± 7.2	7.3 ± 1.1	13.5 ± 2.4	9.5 ± 1.2	7.4 ± 2.0

### Ru-PIP synergizes with Olaparib in lung cancer cells

Next, to access precise quantitative insights of combining Ru-PIP with Olaparib, cells were exposed to increasing concentrations of Ru-PIP alongside sub-cytotoxic concentrations of Olaparib (5 or 10 μM) for 24, 48 and 72 h. Compared to single agents, substantially lower IC_50_ values were observed for the combination treatments (Fig. S2 and Table 1). These results are in accordance with our findings from previous study accessing these combinations in BRCA-wild type breast cancer cell lines^29^. To further our analysis, combination indices (CI) were determined based on established method by Chou and Talalay^30,31^. Synergy (CI < 0.9) was observed at majority of Ru-PIP and Olaparib combinations in A549 and H1975 lung cancer cells at 24 and 48 h incubation times (Fig. 1c). In contrast, most Ru-PIP and Olaparib combinations showed antagonistic effects (CI > 1) in T24 bladder cancer cells. The inconsistent trends seen in T24 cell lines were possibly due to the genetic inconsistencies between these different cell lines which may explain the differences in sensitivity to these combination treatments. Most importantly, mild impact on normal MRC5 cells was observed for most combinations tested, with the overall IC_50_ values obtained are > 54.1 µM (Fig. S2 and Table 1). High cancer selectivity indices were also noted indicating an improved cancer selectivity of these combinations over Ru-PIP alone, particularly in A549 lung cancer cells (Table S1).

### Ru-PIP and Olaparib combination inhibits clonogenic survival and migration ability of lung cancer cells

**Figure 2:**
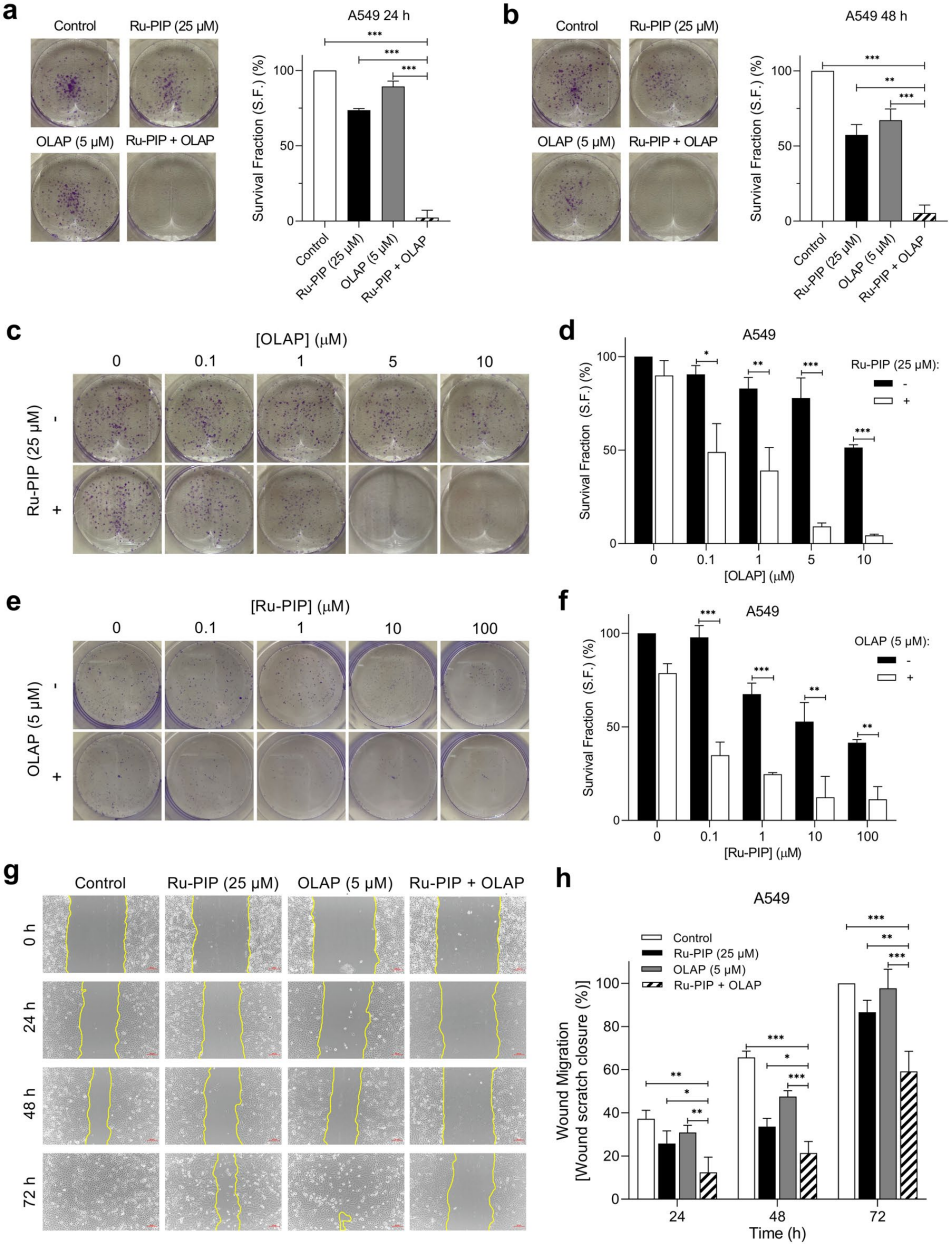
Ru-PIP and Olaparib combination inhibits clonogenic survival and migration ability of lung cancer cells. (**a**) Clonogenic survival assay of A549 lung cancer cells exposed to Ru-PIP (25 µM), Olaparib (5 µM), or both at 24 h treatment. (**b**) Clonogenic survival assay of A549 lung cancer cells, treated as in part a, at 48 h. Mean ± SD of two independent experiments. **P < 0.01, ***P < 0.001 by ANOVA. (**c**) Clonogenic survival assay of A549 cells treated with a concentration gradient of Olaparib with or without Ru-PIP (25 µM), 48 h treatment. (**d**) The survival fractions of A549 cells following treatments were quantified from part c. (**e**) Clonogenic survival assay of A549 cells treated with a concentration gradient of Ru-PIP with or without Olaparib (5 µM), 48 h treatment. (**f**) The survival fractions of A549 cells following treatments were quantified from part e. Mean ± SD of three independent experiments. *P < 0.05, **P < 0.01, ***P < 0.001 by student’s *t*-test. (**g**) Representative images of wound healing assay for A549 cells treated with Ru-PIP (25 µM), Olaparib (5 µM), or both. Cell migration was monitored by optical microcopy at the indicated time points. Scale bar, 50 µm. (**h**) Quantification of percentage of wound closure for cells treated as in part e, as determined by analysis using ImageJ software. Mean ± SD of three independent experiments. *P < 0.05, **P < 0.01, ***P < 0.001 by ANOVA.

The combination effect on the long-term cell survival was assessed using clonogenic survival assay. This determines the ability of a cell to recover post-treatment and reduced colony formation indicates successful induction of reproductive failure of the cells. A549 cells were selected for this purpose based on the superior activity of Ru-PIP in this cell line. Cells were treated with Ru-PIP and Olaparib alone or in combination for 24 and 48 h, and cells were then cultured in compound-free medium until colonies formation which were rendered visible by crystal violet staining. Similar profiles were observed for both 24 and 48 h incubation times in which single-agent treatments had low impact on the loss of clonogenic survival of A549 cells with 48 h survival fractions (S.F.) of > 60% (Fig. 2a,b). Upon treatments with Ru-PIP and Olaparib combinations, however, an almost total loss in the ability of A549 single cells to form colonies was observed. This demonstrates that these combinations were able to inhibit the clonogenic capacity of A549 cells compared to single agent conditions. Furthermore, Ru-PIP was shown to sensitize A549 cells to Olaparib treatments in which substantial reduction in cells survival was observed upon the addition of Ru-PIP to Olaparib-treated cells (Fig. 2c,d). To examine the possibility that PARP inhibition may sensitize A549 cells to Ru-PIP, an additional clonogenic survival assay was carried out in which cells were treated with a concentration gradient of Ru-PIP with or without Olaparib (5 μM). Figure 2e,f shows cells treated with 5 μM Olaparib demonstrate an enhanced sensitivity to Ru-PIP, where cell survival was decreased in comparison to Ru-PIP treatment in isolation. Given the metastatic characteristic of A549 cells, a wound healing assay was carried out to evaluate the effect of Ru-PIP and Olaparib combination on A549 cell migration. The cells were serum-starved prior to treatment initiation to minimize cell proliferation. The scratch for the untreated cells was 50% closed within 24 to 48 h and completely closed (100%) at 72 h, given the fact that A549 cells are highly proliferative and have high migration ability (Fig. 2g,h). As expected, single-agent Olaparib had negligible impact on cell migration. In contrast, 88% of wound closure was observed upon treatment with Ru-PIP alone. Meanwhile Ru-PIP and Olaparib combination showed greater potency in interfering with A549 cells ability to migrate, with 40% reduction in wound closure compared to untreated control. This indicates that the Ru-PIP and Olaparib combination significantly interferes with the motility and migration ability of A549 lung cancer cells.

### Ru-PIP and Olaparib combination enhances apoptotic cell death of lung cancer cells

**Figure 3:**
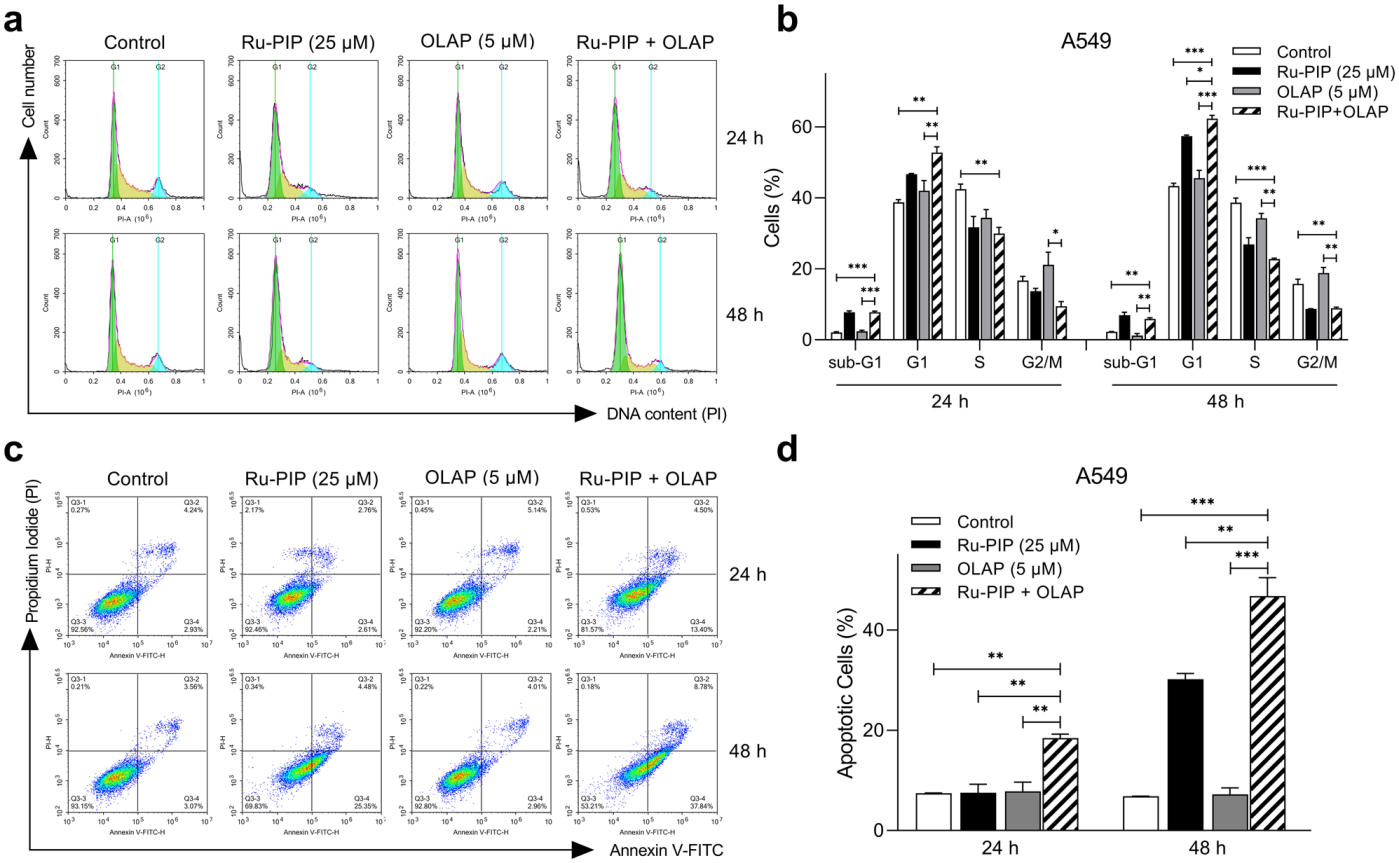
Ru-PIP and Olaparib combination enhances apoptotic cell death. (**a**) DNA histograms showing cell cycle distributions of A549 cells treated with Ru-PIP (25 µM), Olaparib (5 µM) or both for 24 and 48 h. (**b**) Quantification of cell cycle phase distributions from a. (**c**) Annexin V-FITC assay of A549 cells treated with Ru-PIP (25 µM), Olaparib (5 µM) or both for 24 and 48 h. The scatterplots showing the percentage of cells in each quadrant. (**d**) Quantification of apoptotic cells from c. Mean ± SD of two independent experiments. *P < 0.05, **P < 0.01, ***P < 0.001 by ANOVA.

It is of major importance to explore the mechanisms of cytotoxicity that lead to the synergy observed. We first conducted cell cycle analysis by PI staining and flow cytometric analysis to determine whether the synergy observed was due to the alterations in cell cycle progression. Compared to control, single agent Ru-PIP showed small increase in G1 phase (16.1% increase compared to control), meanwhile Olaparib showed comparable cell cycle profile with the untreated control (Fig. 3a,b). Slightly higher accumulation of cells in G1 phase was observed following treatment with Ru-PIP and Olaparib combination compared to single agents alone. Further incubation for 48 h also showed similar cell cycle profile to 24 h treatment where a significant increase in G1 phase cells was observed upon combination treatment than either agent alone. Next, the synergistic combination was tested using trypan blue exclusion assay in which treatment with synergistic Ru-PIP and Olaparib combination showed a time-dependent increase in the percentage of non-viable cells compared to single agents treatments which would be suggestive of cell death (Fig. S3a,b). Following this, an Annexin V-FITC assay was carried out to determine whether the substantial reduction in cell growth upon combination treatment was due to the induction of apoptotic cell death. 24 h treatment with single agents Ru-PIP or Olaparib showed comparable levels of apoptotic cells with untreated control, with longer incubation for 48 h with Ru-PIP alone, increase in apoptotic cells was observed (Fig. 3c,d). Whereas the co-administration of Ru-PIP and Olaparib for 24 h generated a significantly higher percentage of apoptotic cells: a 39.9% increase compared to control. Notably, at longer incubation time of 48 h, increased levels of apoptosis cells were observed in the combination treated group in comparison to 24 h treatment. This assay indicates that the combination induces apoptosis and provides further evidence for Ru-PIP and Olaparib synergism.

### Ru-PIP and Olaparib enhance DSB DNA damage in lung cancer cells

**Figure 4:**
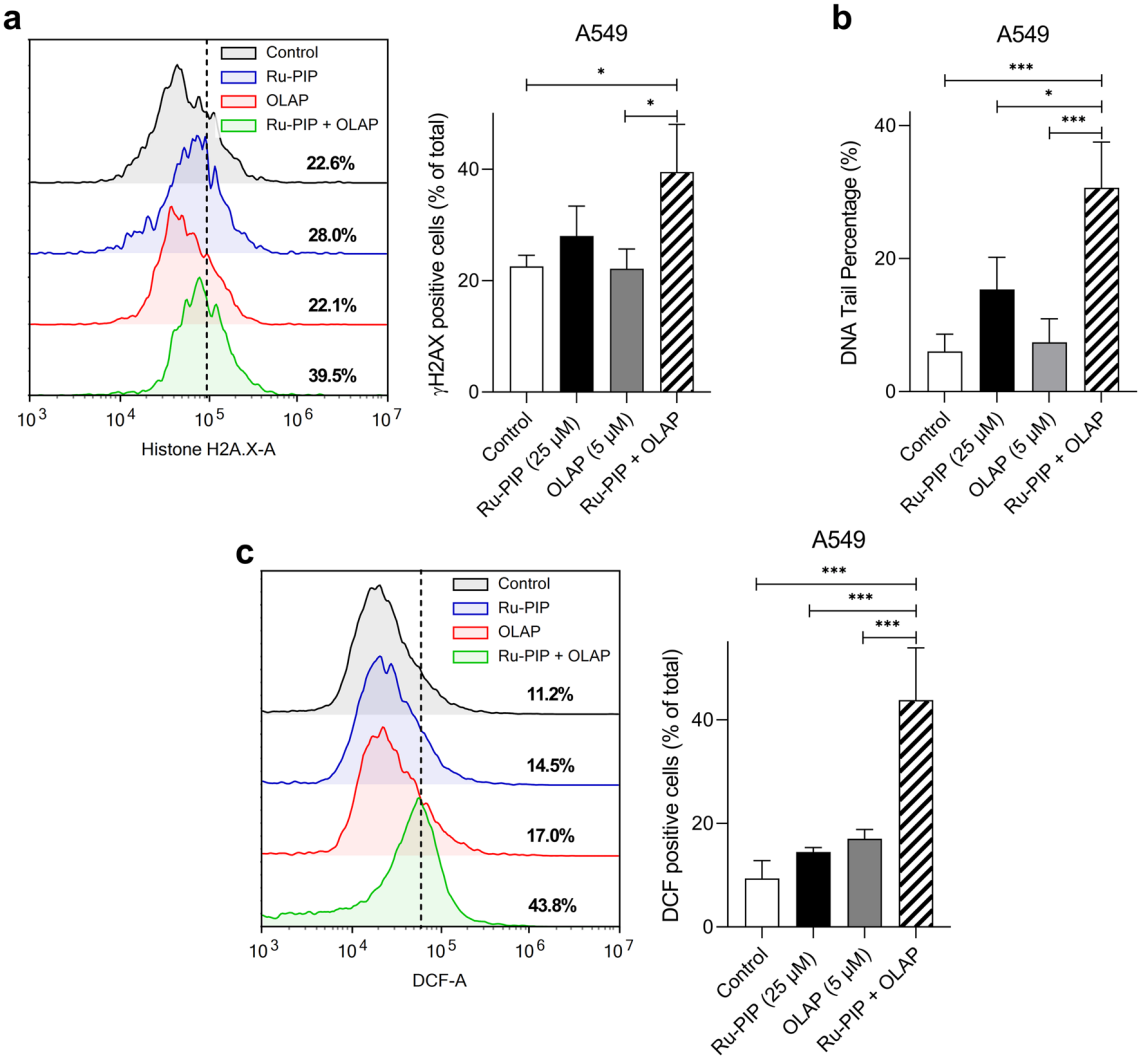
Ru-PIP and Olaparib combination induces DNA damage. (**a**) Left, γH2AX levels upon treatment with Ru-PIP (25 µM), Olaparib (5 µM) or both for 24 h in A549 cells, determined by immunofluorescence and flow cytometry analysis. Right, the percentage of γH2AX positive cells in each population was determined by gating of histograms derived from single-stained cells, as in part a. (**b**) Evaluation of DNA damages upon treatment with Ru-PIP (25 µM), Olaparib (5 µM) or both for 24 h by comet assay. Bar graphs showing the DNA tail percentage (%) in A549 cells, which was used as the parameter for DNA damages. (**c**) Left, histograms showing the fluorescence intensities of 2’,7’-dichlorofluorescein (DCF) upon treatments, as measured by flow cytometry. Right, the ROS levels in A549 cells upon treated with Ru-PIP (25 µM), Olaparib (5 µM) or both for 24 h. Mean ± SD of three independent experiments. *P < 0.05, ***P < 0.001 by ANOVA.

To examine whether the synergy observed was accompanied by increased DNA damage, the levels of $\upgamma$ H2AX (H2AX phosphorylated at Ser139) as an early marker for DNA double-strand breaks (DSBs) was assessed by flow cytometric analysis. Single agent-treated and untreated cells were used for comparison. Single agent treatments for 24 h resulted in comparable $\upgamma$ H2AX levels to those of untreated cells, indicating low DSB damage generated by these compounds as single agents (Fig. 4a). Treatments with the identified combinations showed increase in the proportion of $\upgamma$ H2AX-positive cells (17.0% of total population of cells). An additional alkaline comet assay was carried out to assess the levels of DNA damage. Employing this assay, increased DNA damage was also observed upon combination treatments compared to single agents alone (Fig. 4b). Moreover, sustained DNA damage has been shown to results in the increased levels of reactive oxygen species (ROS). In this work, a 2’,7’-dichlorofluorescein diacetate (DCFDA) probe was used to confirm the generation of ROS which could be oxidized to a green-fluorescent 2’,7’-dichlorofluorescein (DCF) by the intracellular ROS. The fluorescence intensity of DCF is proportional to the amount of peroxide (ROS) produced by the cells. Flow cytometric data showed that treatment with the identified combinations increased ROS generation in comparison to single agents-treated cells and untreated control (Fig. 4c). This confirmed the oxidizing action of these identified combinations in A549 cells and their role in the production of cellular oxidative stress inducing cellular damage and, if damage persists, this results in cell death.

### Ru-PIP and Olaparib combination inhibits the growth of lung cancer spheroid model

**Figure 5:**
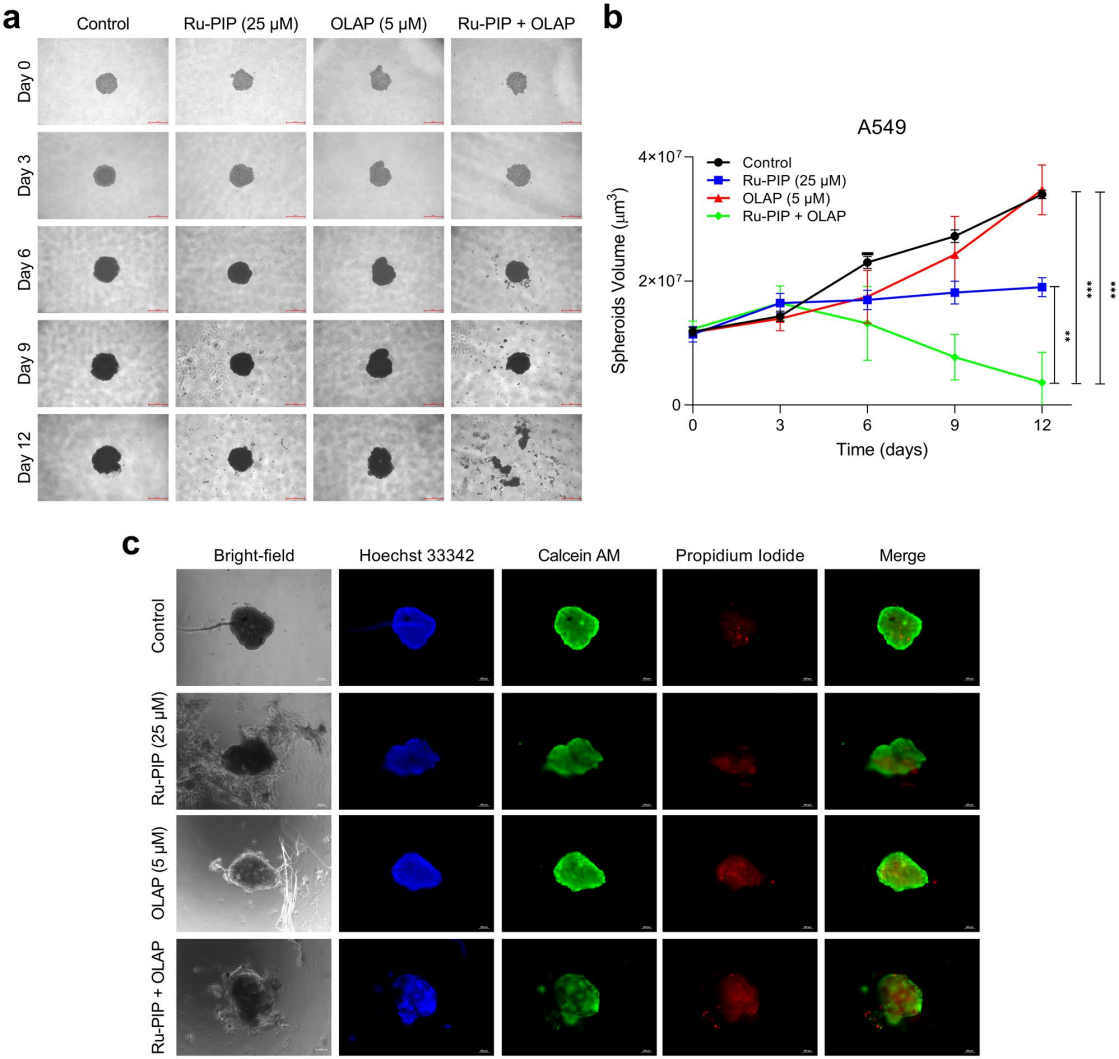
Ru-PIP and Olaparib combination inhibits lung cancer spheroids growth. (**a**) A549 spheroids upon treatments with Ru-PIP (25 µM), Olaparib (5 µM) or both, and images were photographed using light microscope at day 0, 3, 6, 9, and 12 upon treatments initiation. Scale bar = 500 µm. (**b**) Quantification of spheroids volume upon treatments with the stated compounds as in part a. Mean ± SD of eighteen spheroids from three independent experiments. **P < 0.01, ***P < 0.001 by ANOVA. (**c**) Live/dead staining of A549 spheroids upon treatments with Ru-PIP (25 µM), Olaparib (5 µM) or both for 72 h. Live cells indicated by Calcein AM staining (green), dead cells by propidium iodide (PI, red). DNA staining by Hoechst 33342 (blue) provides an indication of total cell number. Bright-field image also included. Scale bar = 100 µm.

For further assessment of the Ru-PIP and Olaparib combination effects in 3D cell culture, we established an A549 spheroid model using hanging drop technique^32,33^. In this manner, A549 cells formed spheroids that managed to grow in diameter in size and volume over the 15-day time frame period (Fig. S4a,b), thereby qualifying them as a viable 3D cell culture model^34,35^. Next, pre-formed spheroids were treated with single agents or Ru-PIP and Olaparib combination, spheroid volume determined, and structure examined. These results show that the structural integrity of the pre-formed spheroids was lost 12 days after treatment initiation with the combination of Ru-PIP and Olaparib (Fig. 5a,b). In contrast to these results, spheroids treated with either Ru-PIP or Olaparib as single agents remained structurally intact following 12 days treatment, although Ru-PIP prevented spheroid growth. We also conducted a live/dead staining experiment to assess cellular viability of the spheroids upon treatment. We observed that treatment with Ru-PIP and Olaparib combination induced more cell death in A549 spheroids compared to single agents treated-spheroids, as indicated by the Calcein AM/PI staining (Fig. 5c). These findings showed that the identified synergistic combination is indeed effective in the more-resistant lung cancer spheroid model.

### Acute zebrafish embryos toxicity test of Ru-PIP and Olaparib combination

**Figure 6:**
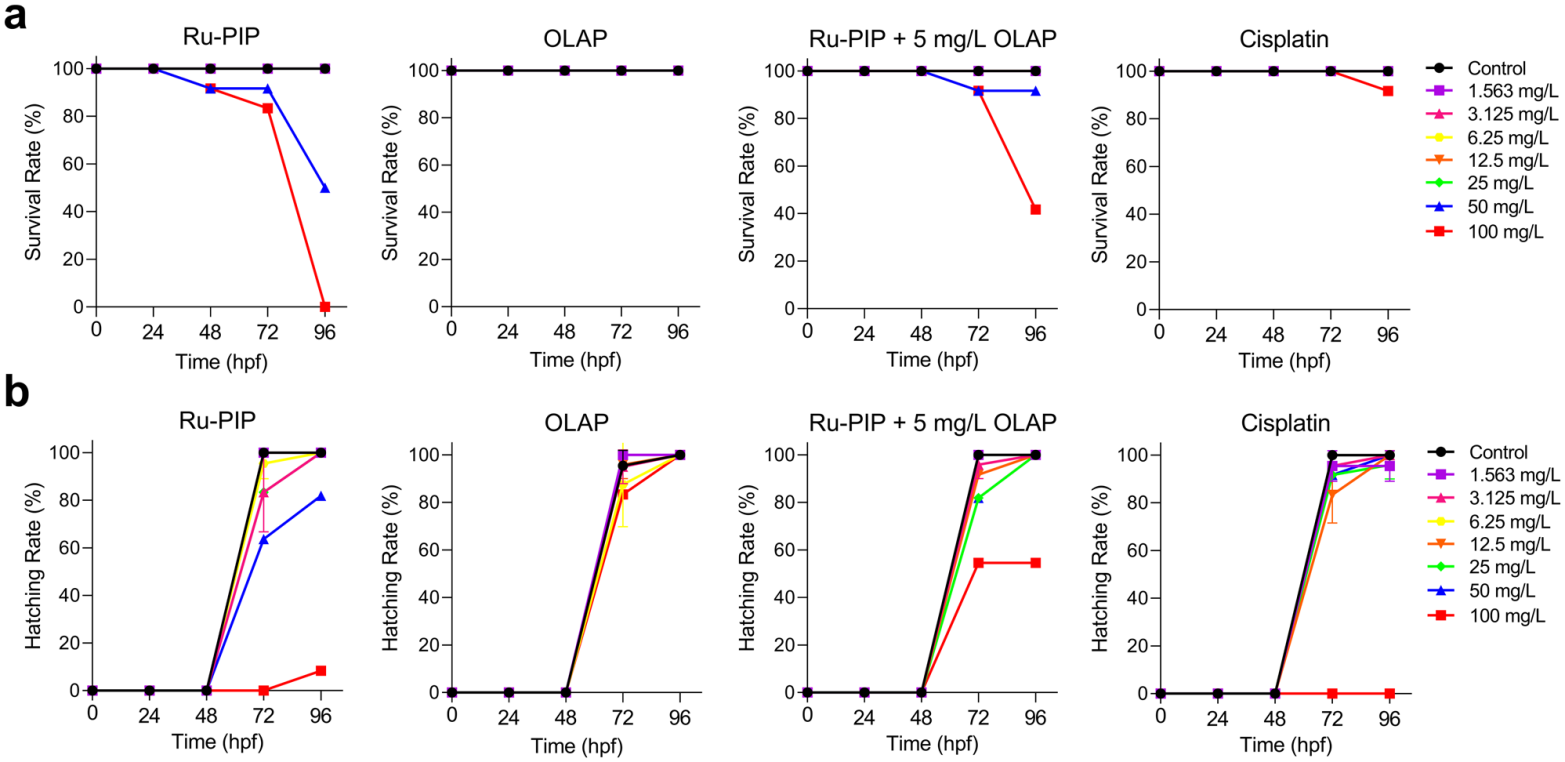
Acute toxicity effects of Ru-PIP without and with Olaparib (5 mg/L) in zebrafish embryos. a) Survival rates and b) hatching rates of zebrafish embryos upon treatment with Ru-PIP, Olaparib, Ru-PIP/Olaparib combination and cisplatin. Subtoxic concentration of Olaparib was used in the combination treatment (5 mg/L). Data expressed as percentage of survival or hatching of twenty-four embryos from two independent experiments. Hpf = hours post fertilisation.

The acute zebrafish embryos toxicity test was carried out to evaluate the toxic effects of the identified combination. With the high degree of homology to mammals, rapid post-fertilization development, small size, and optical transparency, zebrafish embryos have become a widely used model organism for pre-mammalian studies on drug discovery and toxicology evaluation^36–38^. In this study, the embryos were treated with the stated compounds before hatching and thus, the chorion layer shields the embryo until it hatches. Single-agent Ru-PIP showed 96 h half maximal lethal concentration (LC_50_) of 57.5 mg/L (Fig. 6a, Table S2). In comparison to Ru-PIP, Olaparib or cisplatin single agents showed mild toxicity with 96 h LC_50_ value of > 100 mg/L. The increased in embryotoxicity of Ru-PIP at high concentration of 100 mg/L is possibly due to enhanced lipophilicity of Ru-PIP compared to cisplatin as drug absorption through the chorion layer is highly depends on drug’s lipophilicity: cisplatin (partition coefficient, logP = -2.21) and Ru-PIP (log P = 0.403)^28,39^. Notably, the combination of Ru-PIP with subtoxic concentration of Olaparib (5 mg/L) showed higher survival compared to Ru-PIP single-agent with 96 h LC_50_ of 92.8 mg/L. Subsequently, hatching rate upon treatment was also measured in which hatching time normally occurs from 48 hpf (hours post fertilization) to 72 hpf. This is also regarded as an important endpoint toxicity parameter as this will impair their development, inducing other developmental toxicity and eventually, a lethal outcome. Figure 6b shows treatments with 100 mg/L of Ru-PIP or cisplatin prevented almost 100% embryos’ hatching with other works have previously reported the inhibition of zebrafish hatching by cisplatin^40,41^. However, most encouragingly, treatment with 100 mg/L of Ru-PIP in combination with 5 mg/L Olaparib improves hatching rate to 50%.

**Figure 7:**
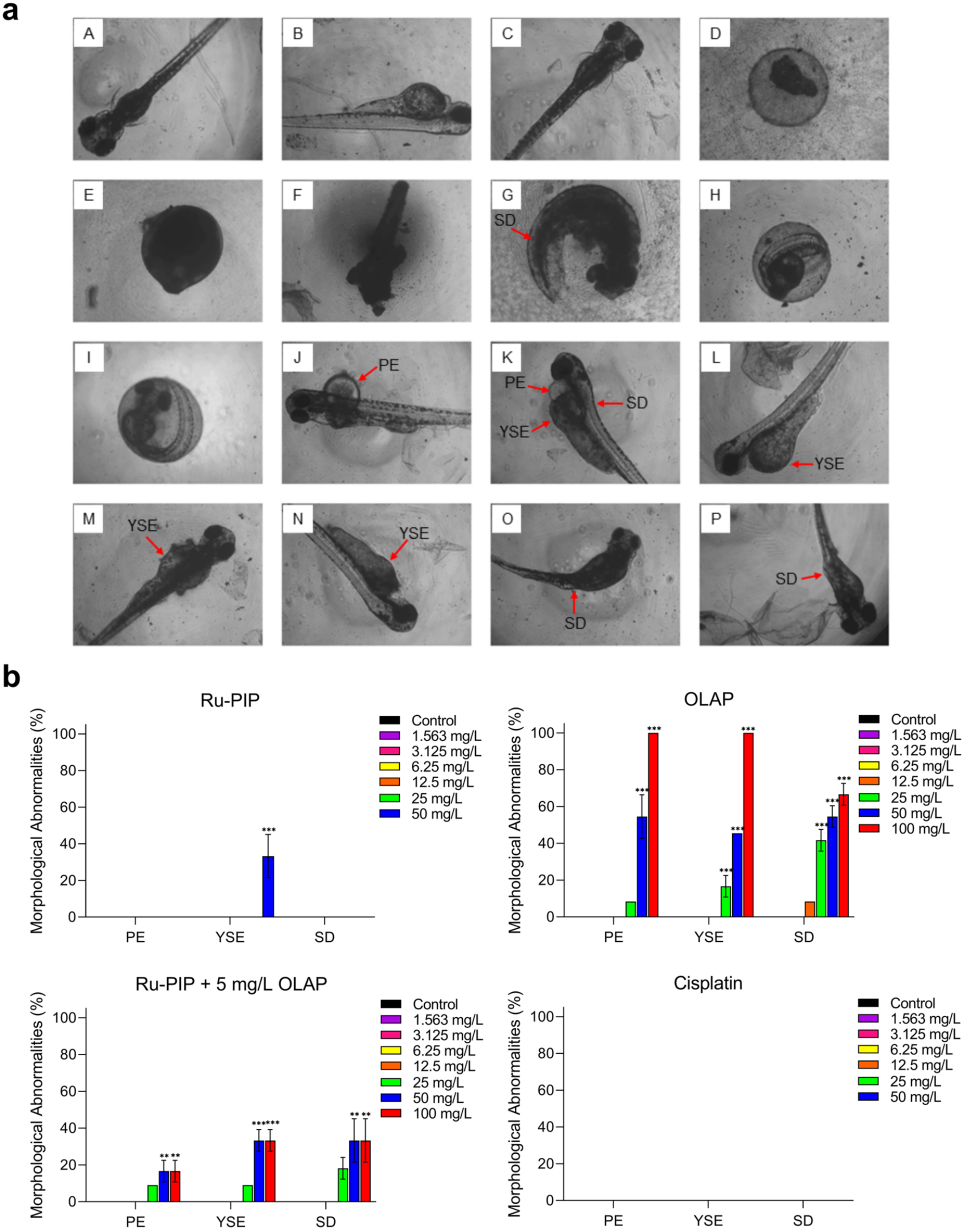
Morphological changes of the zebrafish embryos upon Ru-PIP or Olaparib treatment. (**a**) Morphological assessment of zebrafish embryonic development. (A–C) Normal zebrafish embryonic development at 96 hpf; (D–G) dead or coagulated embryos; (H–I) Non hatching; (J–P) morphological abnormalities; pericardial edema (PE), yolk sac edema (YSE), and spinal deformity (SD). (**b**) Morphological abnormalities of zebrafish embryos upon treatments with Ru-PIP, Olaparib, Ru-PIP/Olaparib combination and cisplatin at 96 hpf. Subtoxic concentration of Olaparib was used in the combination treatment (5 mg/L). Data expressed as percentage of morphological abnormalities observed of twenty-four embryos from two independent experiments. **P < 0.01, ***P < 0.001 compared to control group by ANOVA.

Next, morphological assessment of the embryos upon treatments with the stated compounds was conducted to observe sublethal effects including pericardial edema, yolk sac edema and spinal deformity. The embryos were not accessed for morphological changes upon treated with 100 mg/L of Ru-PIP or cisplatin as the embryos did not survive or they remain unhatched. Notably, upon treated with > 25 mg/L of Olaparib, the embryos showed substantial morphological abnormalities of pericardial edema, yolk sac edema and spinal deformity (Fig. 7a,b). Treatment with 50 mg/L of Ru-PIP alone led to 33% of the embryos showing yolk sac edema, meanwhile treatment with cisplatin at any concentration did not show any morphological changes. Morphological changes were also observed in embryos treated with > 50 mg/L of Ru-PIP in combination with subtoxic concentration of Olaparib (yolk sac edema and spinal deformity, 33%; and pericardial edema, 16%). Overall, although Ru-PIP single-agent at high concentrations showed lower survival in comparison to cisplatin, its combination with Olaparib showed higher survival than Ru-PIP alone and hatching rate was also improved in comparison to both cisplatin and Ru-PIP alone. The basis for this surprising finding is unknown, however, as the combined treatment of Ru-PIP and Olaparib exhibited lower toxicity than the single-agent Ru-PIP, further genotoxicity studies using zebrafish embryos model should be conducted to elucidate the suppressive effect of PARP inhibition in Ru-PIP-treated zebrafish embryos.

## Discussion

Although platinum-based drugs exhibit potent anti-cancer activity in NSCLC patients, long response times can lead to the rapid development of chemoresistance and may result in severe side effects. Therefore, current platinum-based therapy for NSCLC remains insufficient. Hence, the key to enhanced treatment for NSCLC patients are through both development of new anti-cancer drug and novel combination therapy^42^. In recent years, RPCs have emerged as potential anti-cancer candidates as alternative to current platinum-based drugs^43^. Whilst the majority of studies have examined RPCs as single agents, they have been used alongside light as photosensitizers for photodynamic therapy (PDT) and also explored as radiosensitizers for ionizing radiation (IR)^22,44,45^. However, the combination of RPCs with PARPi remains an unexplored strategy, although the potential for this is evidenced by the fact PARPi single agents treatments have been widely examined including in both NSCLC and SCLC^46,47^.

In this study, we demonstrate favorable synergy between Ru-PIP and Olaparib in lung cancer cells (Figs. 1 and 2), further affirming that this combination confers activity in various cancer cell lines. High cancer selectivity of the identified combination in comparison to their single agents provide information about their potential for pre-clinical in vivo and clinical tests. Mechanistic studies revealed that the synergy observed was resulted from increased DNA DSB damage and ROS levels which subsequently lead to cell death via apoptosis (Figs. 3 and 4). These results are consistent with previous work demonstrating the synergy between Ru-PIP and Olaparib on breast cancer cells and the ability of Ru-PIP to sensitizes BRCA-wild type cells to Olaparib treatment^29^. Other works have examined the combination of Olaparib with other DNA damaging agents, for instance, the combination of Olaparib and cisplatin which was assessed in several cancer cells^48,49^. Compared to single agents alone, Olaparib and cisplatin combination showed enhanced apoptotic cell death which is accompanied by sustained DSBs. In addition to this, the combination of Olaparib with carboplatin also showed synergy, with further mechanistic studies revealed that the synergy observed was resulted from enhanced DNA damage^50,51^. In contrast to these platinum-based drugs, single-agent Ru-PIP induces high levels of DNA replication stress without inducing DSBs and/or apoptotic cell death. This may be advantageous considering that majority of other DNA damaging agents explored in combination with PARPi including cisplatin and carboplatin showed substantial levels of cytotoxic DNA damage and thus, utilizing Ru-PIP alongside PARPi may be able to reduce unfavourable mechanistic overlap with PARPi. Furthermore, since pharmacological inhibitors may have off-target effects, another study demonstrating that PARP1 and PARP2 which play a critical role in the stabilization of replication fork should be explored^14^. By showing whether the combination of Ru-PIP and the genetic inhibition of PARP1 and PARP2 (by PARP1/2 knockdown) would exhibit synergy as well will confirm that the observed effects are indeed be attributed to PARP1 inhibition.

In addition to cytotoxicity and drug penetration, evaluation of drug combination effects in spheroids model can also provide additional insight, as 3D structure often better recapitulate the tissue microenvironment in vivo by mimicking the complexity and heterogeneity of cellular organization in clinical tumors^51–53^. Here, we demonstrate that the combination of Ru-PIP and Olaparib is able to inhibit the growth of the more-resistant lung cancer spheroids model (Fig. 5). We further assessed the toxicity effects of the identified combination and their single agents in the zebrafish embryos model. Compared to cisplatin, single-agent Ru-PIP at high concentrations showed low survival with substantial morphological changes of the zebrafish embryos were observed (Figs. 6 and 7). However, at lower concentration of < 25 mg/L, the embryos survive without any morphological defects indicating that the concentration applied in the in vitro studies is within the safety limit. Nevertheless, Ru-PIP in combination with Olaparib showed higher survival than Ru-PIP alone. And notably, although both cisplatin and Ru-PIP single-agent at high concentration prevented 100% embryos hatching, improves hatching rate was observed upon combination treatment. This demonstrates that the synergistic combination of Ru-PIP and Olaparib may potentially reduce side effects observed in single-agent therapy. Despite the promising findings presented within this study, additional mutagenicity and/or genotoxicity studies should be conducted to further access the safety limit of Ru-PIP and its combination with Olaparib in order to determine any adverse effects resulting from off-target effects exerted by these agents and explore the therapeutic window for this specific combination^26,54^ Collectively, the systematic approach used in this drug combination study can be utilized by researchers in the drug combination field as it enhances the biological relevance of the data and the additional acute toxicity test on zebrafish embryos model can preclinically determine the safety limit of newly discovered therapeutic agents, prior to in vivo studies.

## Methods

### Materials

[Ru(dppz)_2_(PIP)^2+^ (Ru-PIP) was synthesized and purified as previously reported^28^. Olaparib (OLAP) was purchased from MedChemExpress and cisplatin from SigmaAldrich. All other chemicals were purchased from SigmaAldrich, ThermoFisher Scientific, unless stated otherwise. Antibodies for p-histone H2A.X (Ser139) were purchased from Cell Signaling Technology (CST). Antibodies for Alexa-Fluor 488 conjugated secondary antibodies were purchased from Abcam.

### Stock preparations

Stock solutions of Ru-PIP and Olaparib were prepared at 100 mM by dissolving in 100% dimethyl sulfoxide (DMSO) and stock solution of cisplatin was prepared at 2 mM by dissolving in 1X phosphate buffer saline (PBS). All stock solutions were stored at − 20 °C and further diluted with cell culture media or 1X PBS before use. Final DMSO concentration for cell culture was kept below ≤ 0.1%.

### Cell lines

A549 lung cancer, H1975 lung cancer, T24 bladder cancer and MRC5 normal human lung fibroblast cell lines were obtained from ATCC and cultured in DMEM supplemented with 10% fetal bovine serum (FBS) and 1% penicillin/streptomycin (P/S) antibiotic. All cell lines were cultured as monolayers and maintained in CO_2_ incubator at 37 °C under a humidified atmosphere containing 5% CO_2_.

### MTT assay

Cells were seeded in 96-well plates at 5 × 10^3^ cells/well and allowed to adhere overnight. For single-agent treatment, cells were treated with concentration gradient of Ru-PIP, Olaparib and cisplatin (3.125–100 µM). For drug combination treatment, cells were treated with concentration gradient of Ru-PIP (3.125–100 µM) in combination with Olaparib (5 or 10 µM). After 24, 48 or 72 h incubation, 0.5 mg/ml thiazolyl blue tetrazolium bromide (MTT) dissolved in 1X PBS was added to each well and cells were incubated at 37 °C for 4 h. 100 µl of DMSO was added to each well to solubilize the reduced purple formazan crystals formed. Next, the absorbance at 570 nm (630 nm as reference wavelength) was measured using a microplate reader. The half maximal inhibitory concentration (IC_50_) for each compound was determined using GraphPad Prism software. Additionally, selectivity indices (SI) were determined by the ratio between IC_50_ of normal cells and that of cancer cells.

### Drug interaction analysis

Based on the data obtained from MTT assay, combination indices (CI) were determined using Calcusyn and CompuSyn software (Biosoft, Cambridge, UK). This is based on the established drugs combination method by Chou and Talalay^30,31^. CI values indicate the type of drugs interaction in which CI < 0.9 indicates synergism, CI = 0.9–1.0 indicates additive and CI > 1.0 indicates antagonism. GraphPad Prism software was used to generate a three-color scale based on CI values obtained, where synergism is represented by green, additive by yellow, and antagonism by red in which the colors of each CI value were interpolated in between these constraints accordingly.

### Clonogenic survival assay

Lung cancer cells were seeded in 6-well plates at 1 × 10^3^ cells/well and allowed to adhere overnight. Cells were treated with Ru-PIP (25 µM), Olaparib (5 µM) or both for 24 and 48 h. For the gradient assay, cells were treated with increasing concentration of Olaparib (0, 0.1, 1, 5 and 10 µM) with and without Ru-PIP (25 µM) for 48 h. Cells were also treated with increasing concentration of Ru-PIP (0, 0.1, 1, 10 and 100 µM) with and without Olaparib (5 µM) for 48 h. After treatment, solutions were removed, and cells were grown in compound-free medium for 7–10 days for colony formation. Cells were washed with ice-cold 1X PBS, fixed with ice-cold 100% methanol (20 min, on ice) and stained visible with 0.5% crystal violet solution (20 min, room temperature (RT)). The staining solution was washed away under running tap water and images were photographed with a digital camera. Using ImageJ software, individual colonies were counted, and the survival fraction (S.F.) for each sample was determined (normalized to controls).

### Trypan blue exclusion assay

Lung cancer cells were seeded in 6-well plate at 1 × 10^5^ cells/well and allowed to adhere overnight. Cells were treated with Ru-PIP (25 µM), Olaparib (5 µM) or both for 24 and 48 h. After treatment, cells were harvested, washed with 1X PBS twice and concentrated via centrifugation. 0.1% trypan blue solution dissolved 0.25 ml 1X PBS was added to each sample and mixture was incubated for at least 3 min at RT. Non-viable (blue) and viable (non-blue) cells were counted using hemocytometer under light microscope. A minimum of 200 cells were counted for each sample and the percentage of trypan blue positive and negative cells were determined.

### Wound healing assay

Lung cancer cells were seeded at density of 5 × 10^5^ cells/well in a 6-well plate and were incubated in the incubator until a confluent monolayer of cells formed. After serum-starved for 4 h, a straight scratch was made in each well with a sterile 200 µl pipette tip, solution was removed, and each well was washed with 1X PBS twice. Cells were treated with Ru-PIP (25 µM), Olaparib (5 µM) or both. Cells migration in the wound scratch area was photographed at 0, 24, 48 and 72 h timepoints. Images were photographed at 10 × magnification using a camera attached microscope and were analyzed using ImageJ software. The percentage of wound scratch closure was determined by measuring the reduction in the wound area at each time point by comparing to 0 h (100%).

### Cell cycle analysis

Lung cancer cells were seeded in 6-well plate at density of 1 × 10^5^ cells/well and allowed to adhere overnight. Cells were treated with Ru-PIP (25 µM), Olaparib (5 µM) or both. After 24 or 48 h incubations, cells were harvested, washed with 1X PBS twice, and fixed with ice-cold 70% ethanol at least overnight at − 20 °C. The fixed cells were collected by centrifugation at 1,000 rpm for 5 min and the resulting pellets were washed with 1X PBS twice. The samples were resuspended in 500 µl PBS and treated with 5 µl RNase A solution (10 mg/ml) for 15 min at RT. After incubation, the samples were stained with 2 µl of propidium iodide (PI) solution (5 mg/ml) at RT, in the dark. Samples were acquired, and cell cycle distribution was analyzed using NovoCyte flow cytometer and NovoExpress software. For each sample, a minimum of 10,000 cells were counted and analyzed.

### Apoptosis annexin V-FITC assay

Lung cancer cells were seeded in 6-well plate at 1 × 10^5^ cells/well and allowed to adhere overnight. Cells were treated with Ru-PIP (25 µM), Olaparib (5 µM) or both. After 24 or 48 h incubations, cells were harvested, washed with 1X PBS twice and 500 µl of 1X binding buffer was added to the samples. After, the samples were incubated with 5 µl Annexin V-FITC (Invitrogen) for 20 min at RT. Prior to flow cytometric analysis, 5 µl of PI was added. Samples were acquired was analyzed using NovoCyte flow cytometer and NovoExpress software. For each sample, a minimum of 10,000 cells were counted and analyzed.

### Determination of reactive oxygen species (ROS) levels

Lung cancer cells were seeded in 6-well plate at density of 1 × 10^5^ cells/well and allowed to adhere overnight. Cells were treated with 10 μM of 2’,7’-dichlorofluorescein diacetate (DCFDA) in serum-free growth media for 30 min at 37 °C in the dark. After incubation, the DCFDA solution was removed, cells were washed with 1X PBS twice and cells were treated with Ru-PIP (25 µM), Olaparib (5 µM) or both for 24 h. After incubation, cells were harvested, washed with 1X PBS twice and resuspended in 1X PBS. Intensity of the formed 2′7’-dichlorofluorescein (DCF) as a result of carboxy-DCFDA hydrolysis by intracellular ROS were analyzed using NovoCyte flow cytometer and NovoExpress software at an excitation and emission wavelength of 488 nm and 525 nm, respectively. For each sample, a minimum of 10,000 cells were counted.

### γH2AX immunostaining

Lung cancer cells were seeded at 3 × 10^5^ cells/well 6-well plates and allowed to adhere overnight. Cells were treated with Ru-PIP (25 µM), Olaparib (5 µM) or both for 24 h. After treatment, cells were harvested, washed with 1X PBS twice and fixed with 4% paraformaldehyde (PFA) for 15 min at RT. After fixation, fixed cells were centrifuged, and the resulting cell pellets were washed with 1X PBS. Cells were resuspended in 500 µL of 1X PBS, permeabilized by adding ice-cold 100% methanol slowly to pre-chilled cells, to a final concentration of 90% methanol, and cells were left for at least 10 min on ice. Cells were washed in excess 1X PBS, resuspended in 100 µL diluted primary antibody (γH2AX) and incubated for 1 h at RT. Cells were then washed with antibody dilution buffer, resuspended in 100 µL of diluted fluorochrome-conjugated secondary antibody and incubated for 30 min at RT, in the dark. Thereafter, cells were washed with antibody dilution buffer and were resuspended in 500 µL 1X PBS. Samples were acquired and analysed with a NovoCyte flow and NovoExpress software. For each sample, a minimum of 10,000 cells were counted.

### Alkaline comet assay

Lung cancer cells were seeded in 24-well plate at density of 1 × 10^5^ cells/well and allowed to adhere overnight. Cells were treated with Ru-PIP (25 µM), Olaparib (5 µM) or both for 24 h. After incubation, cells were harvested, resuspended in ice-cold 1X PBS and 20 µL of cell suspension was mixed with 120 µL of 1% low melting agarose (1/10 ratio; V/V). Immediately thereafter, 80 µL of the cell suspension were dropped onto the pre-coated (1% normal melting agarose) agarose slides, covered with coverslips and the slides were cooled for 15 min at 4 °C, in the dark. After that the coverslips were removed, the slides were immersed in pre-chilled lysis buffer (2.5 mM NaCl, 100 mM Na_2_EDTA, 100 mM Tris–HCl and 1.6 g NaOH; pH 10) and incubated 2 h at 4 °C, in the dark. The slides were then immersed in pre-chilled alkaline solution (1 mM Na_2_EDTA and 300 mM NaOH; pH > 13) and incubated 1 h at 4 °C, in the dark. Thereafter, electrophoresis was conducted in a chamber filled with pre-chilled alkaline electrophoresis solution (300 mM NaOH and 1.0 mM EDTA; pH > 13), under standard conditions (22 V; 300 mA; 1 V/cm) for 30 min in the dark. The slides were then neutralized with neutralization buffer (0.4 M Tris–HCl, pH 7.5) for 10 min, washed with ice-cold water (2 × 10 min), fixed in ice-cold 70% ethanol for 5 min and air dried for 15–30 min. Finally, the slides were stained with 300 µL of 5 µg/mL Hoechst 33,342 solution in PBS for 30 min at RT in the dark, and the slides were imaged using fluorescence microscope. At least 50 cells were analyzed per sample and in total, three cultures were performed per treatment. The percentage of DNA in the tail was used as a parameter of DNA damage.

### Spheroids growth studies

Spheroids establishment was attempted for A549 lung cancer cells using hanging drop method. 20 µL of cell suspension containing 2000, 4000, 6000, 8000 and 10,000 cells were dropped onto the surface of petri dish lid and incubated at 37 °C, such that each drop will contain single spheroid. After three days of incubation, each spheroid formed was transferred onto agarose-coated (50 µL, 0.6%) 96-well plates containing 80 µL growth media and further incubated for 15 days at 37 °C, such that each well contained a single spheroid. Formation of spheroids was observed and imaged every three days using a light microscope with a 4 × objective lens connected to a digital camera. During growth, 50% of the media was replaced every three days. The growth in volume of each spheroid was determined according to a published method^35^. At least six spheroids were grown per seeding density for each independent experiment.

### Spheroids growth inhibition studies

Spheroids were grown as described initially. Initial cell seeding density was chosen such that each spheroid reached a diameter of approximately 400 μm after three days (4,000 cells/well for A549 cells). After spheroids formation, spheroids were incubated with Ru-PIP (25 µM), Olaparib (5 µM) or both for a period of 12 days. The structural integrity of spheroids upon treatment was observed and imaged for every three days using a light microscope with a 4 × objective lens connected to a digital camera, and the volume of each spheroid was measured according to a published method^35^. Prior to imaging, 50% of treatment-containing medium was replaced (every three days). At least six spheroids were grown per treatment for each independent experiment.

### Spheroids live/dead staining

Spheroids were grown as described initially. The preformed spheroids were transferred into agarose-coated (172 µL, 0.6%) 48-well plates. Spheroids were incubated with Ru-PIP (25 µM), Olaparib (5 µM) or both by replacing 50% of the medium with treatment-containing media. Following 72 h incubation, half of the culture media was replaced with staining solutions at 2X of their final concentrations for 30 min at 37 °C, in the dark. The final concentrations used were 1 µM, 5 µg/mL and 2 µg/mL for Calcein AM, Hoechst 33342 and propidium iodide (PI) in 1X PBS, respectively. Next, the spheroids were washed with 1X PBS and fixed with 4% PFA for 30 min at RT. After fixation, spheroids were washed with 1X PBS twice and the triple-stained spheroids were imaged using a fluorescence microscope to evaluate cellular viability. At least six spheroids were grown per treatment for each independent experiment.

### Acute toxicity test on zebrafish embryos

Acute zebrafish embryo toxicity test was conducted according to the guidelines from the Organization for Economic Cooperation and Development (OECD) Test No. 236: Fish Embryo Toxicity (FET) test^55^. All procedures adhered strictly to the guidelines for care and use of Animal Biochemistry & Biotechnology Laboratory, Faculty of Biotechnology and Biomolecular Sciences, Universiti Putra Malaysia (UPM) which has been approved by Institutional Animal Care and Use Committee (IACUC) of UPM (UPM/IACUC/AUP No. R059/2018). The newly fertilized eggs at less than 1 hpf (hours post fertilization) were collected, washed with deionized water and incubated at RT (28 ± 1 °C) in Danio-SprintM embryo media. Embryos were transferred into 96-well plates (one embryo/well) and were exposed to concentration gradients (1.56 to 100 mg/L) of Ru-PIP, Olaparib, cisplatin or Ru-PIP in combination with 5 mg/L Olaparib. Untreated control consisting of Danio-SprintM embryo media containing 0.1% DMSO. 24 embryos were used in total for each concentration per treatment of two independent experiments (*n* = 24). The zebrafish embryos development was observed and imaged using a light microscope attached to a digital camera at 24, 48, 72 and 96 hpf (hours post fertilization). Four lethal endpoints were evaluated including coagulated embryos, lack of somite formation, non-detachment of the tail and lack of heartbeat. All these characteristics were recorded every 24 hpf, except heartbeat, which is visible after 48 hpf. The concentration of compound(s) which causes death of 50% of zebrafish embryos (LC_50_) were determined by using GraphPad Prism software. Hatching which is also considered as a sensitive endpoint toxicity parameter was also observed in which hatching normally occurs at 48 to 72 hpf. Additionally, the morphological changes including pericardial edema, yolk sac edema and spinal deformity after exposure to the stated compounds were observed and imaged.

### Statistical analysis

Statistical analysis of the data was carried out using GraphPad Prism software in which the data obtained was analyzed using student’s *t*-test or one-way analysis of variance (ANOVA). The differences between the groups were considered significant when *P* values generated were less than 0.05.

## Supplementary Information

Supplementary Information.

## Data Availability

Any data generated from these studies is available from the corresponding author upon reasonable request.
