# Supplementary material for: Synergy of ruthenium metallo-intercalator, [Ru(dppz)2(PIP)]2+, with PARP inhibitor Olaparib in non-small cell lung cancer cells
Source: Sci Rep. 2023 Jan 26;13:1456. doi: 10.1038/s41598-023-28454-x (PMC9879939; doi:10.1038/s41598-023-28454-x)
Supplement: Supplementary file 1 — Supplementary Information. [file 41598_2023_28454_MOESM1_ESM.docx]

**Synergy of ruthenium metallo-intercalator, [Ru(dppz)_2_(PIP)]^2+^, with PARP inhibitor Olaparib in non-small cell lung cancer cells**

Nur Aininie Yusoh^a^, Suet Lin Chia^a,b^, Norazalina Saad^a^, Haslina Ahmad^a,c^*, and Martin R. Gill^d^*

**^a^**UPM-MAKNA Cancer Research Laboratory, Institute of Bioscience, Universiti Putra Malaysia, 43400 UPM Serdang, Selangor, Malaysia

^b^Department of Microbiology, Faculty of Biotechnology and Biomolecular Science, Universiti Putra Malaysia, 43400 UPM Serdang, Selangor, Malaysia

^c^Department of Chemistry, Faculty of Science, Universiti Putra Malaysia, 43400 UPM Serdang, Selangor, Malaysia

^d^Department of Chemistry, Faculty of Science and Engineering, Swansea University, Swansea, UK

email: [m.r.gill@swansea.ac.uk](mailto:m.r.gill@swansea.ac.uk) or [haslina_ahmad@upm.edu.my](mailto:haslina_ahmad@upm.edu.my)

**Supplementary Information**

**Table S1.** Selectivity Index (SI) = IC_50_ normal cells/IC_50_ cancer cells. ND = not determined.

| Compound | Selectivity Index (SI) | | | | | | | | |
| --- | --- | --- | --- | --- | --- | --- | --- | --- | --- |
|  | A549 | | | H1975 | | | T24 | | |
|  | 24 h | 48 h | 72 h | 24 h | 48 h | 72 h | 24 h | 48 h | 72 h |
| Ru-PIP | >2.8 | 4.6 | >8.7 | ND | 0.9 | >1.6 | >2.2 | 4.0 | >6.3 |
| Olaparib | ND | ND | ND | ND | ND | >1.3 | ND | ND | ND |
| Ru-PIP + 5 µM Olaparib | 4.7 | 14.1 | 10.0 | <0.6 | 1.2 | 2.2 | 1.6 | 4.4 | 2.3 |
| Ru-PIP + 10 µM Olaparib | 6.3 | 11.5 | 8.9 | 1.0 | 2.1 | <0.6 | 1.4 | 4.2 | 4.2 |
| Cisplatin | 2.0 | 1.9 | 2.5 | 0.5 | 0.4 | 0.5 | 0.2 | 0.4 | 1.0 |

**Table S2.** The half maximal lethal concentration (LC_50_) of the stated compound(s) upon 96 h treatments on zebrafish embryos.

| Compound | 96 h LC_50_ (mg/L) |
| --- | --- |
| Ru-PIP | 55.5 |
| Olaparib | >100 |
| Ru-PIP + 5 mg/L Olaparib | 92.8 |
| Cisplatin | >100 |


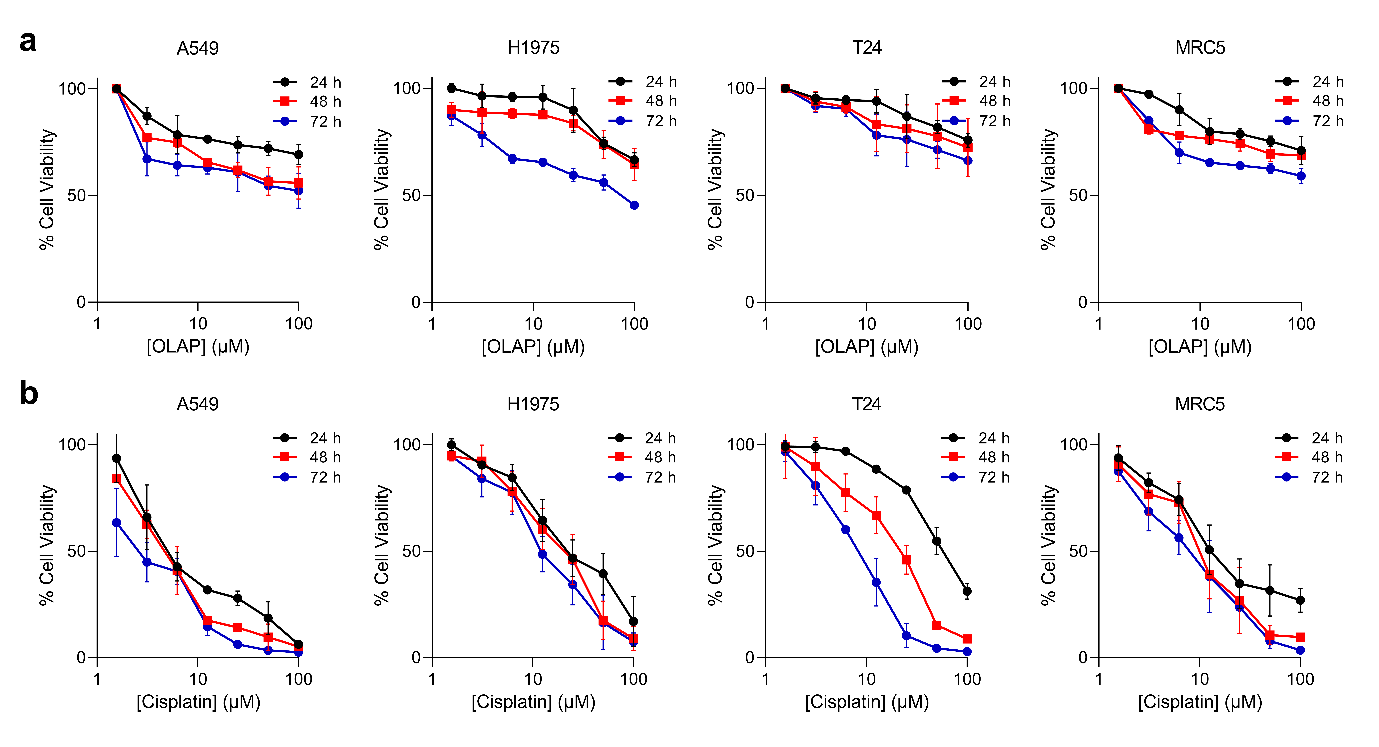


**Figure S1.** Olaparib and cisplatin led to dose- and time-dependent decreases in cell viability of several cancer cells. a) Cell viability of A549 lung cancer cells, H1975 lung cancer cells, T24 bladder cancer cells and MRC5 normal lung cells upon treatment with concentration gradient of Olaparib for 24, 48 and 72 h, as determined by MTT assay. c) Cell viability of cells upon treated with cisplatin as described in part a. Mean ± SD of three independent experiments.


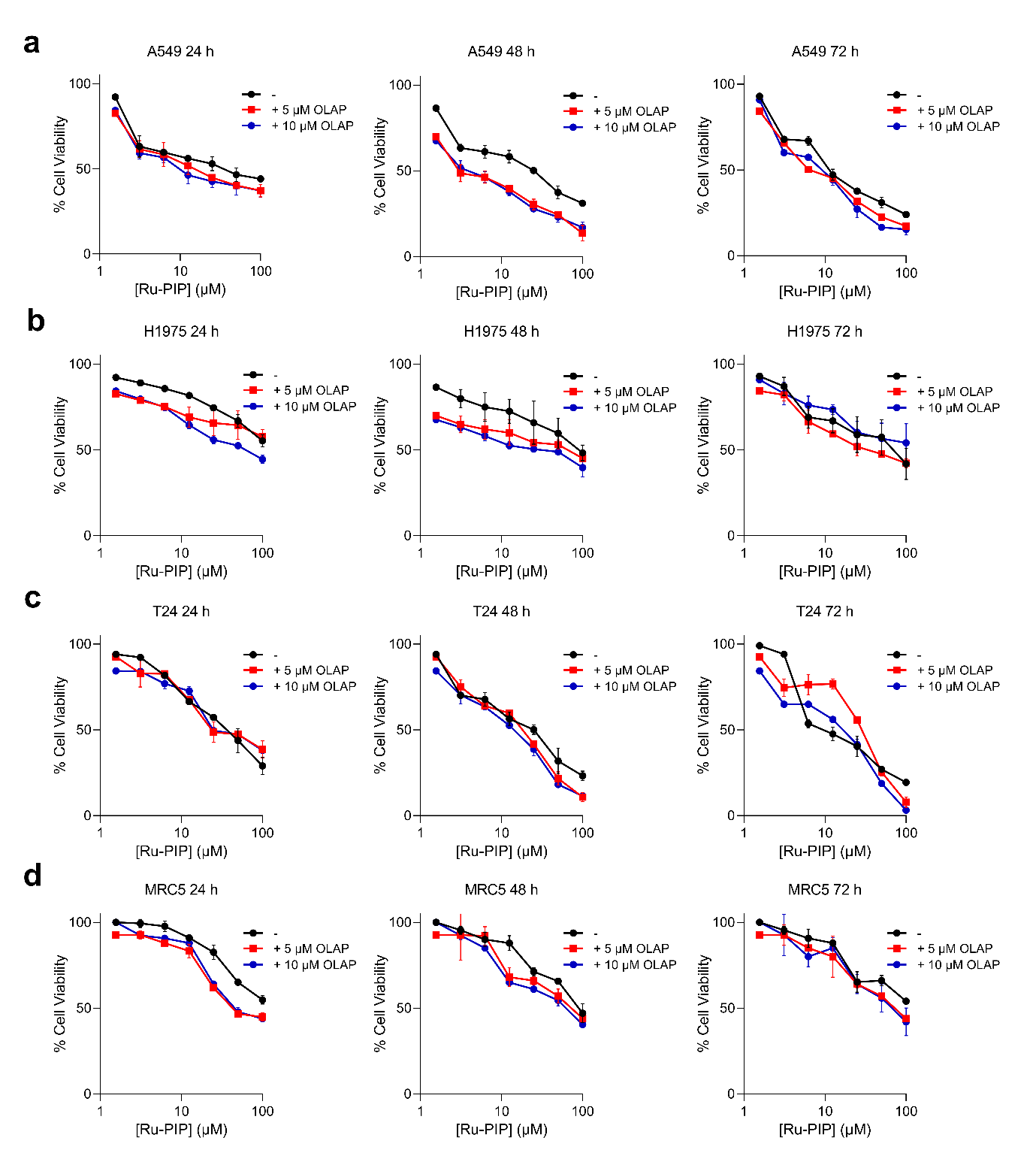


**Figure S2.** Ru-PIP and Olaparib combination showed reduction in cell viability of lung cancer cells compared to Ru-PIP single-agent alone. Cell viability of a) A549 lung cancer cells, b) H1975 lung cancer cells, c) T24 bladder cancer cells and d) MRC5 normal lung cells upon treatment with concentration gradient of Ru-PIP alone or in combination with Olaparib for 24, 48 and 72 h, as determined by MTT assay. Sub-cytotoxic concentration of Olaparib (5 or 10 μM) was used in the combination treatment. Mean ± SD of three independent experiments.


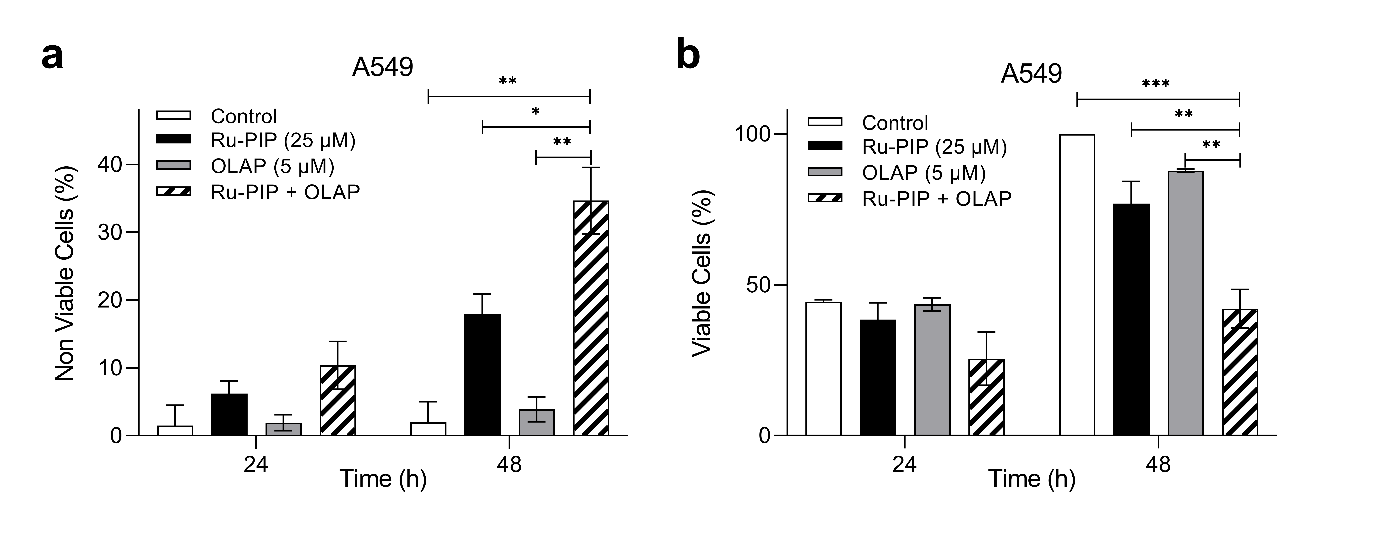


**Figure S3.** Ru-PIP and Olaparib combination showed time-dependent increase in the percentage of non-viable cells of A549 cells. a) Non-viable and b) viable A549 cells upon treatment with Ru-PIP (25 µM), Olaparib (5 µM), or both for 24 and 48 h, as determined by trypan blue exclusion assay. Mean ± SD of two independent experiments. **P* < 0.05, ***P* < 0.01, ****P* < 0.001 by ANOVA.

**
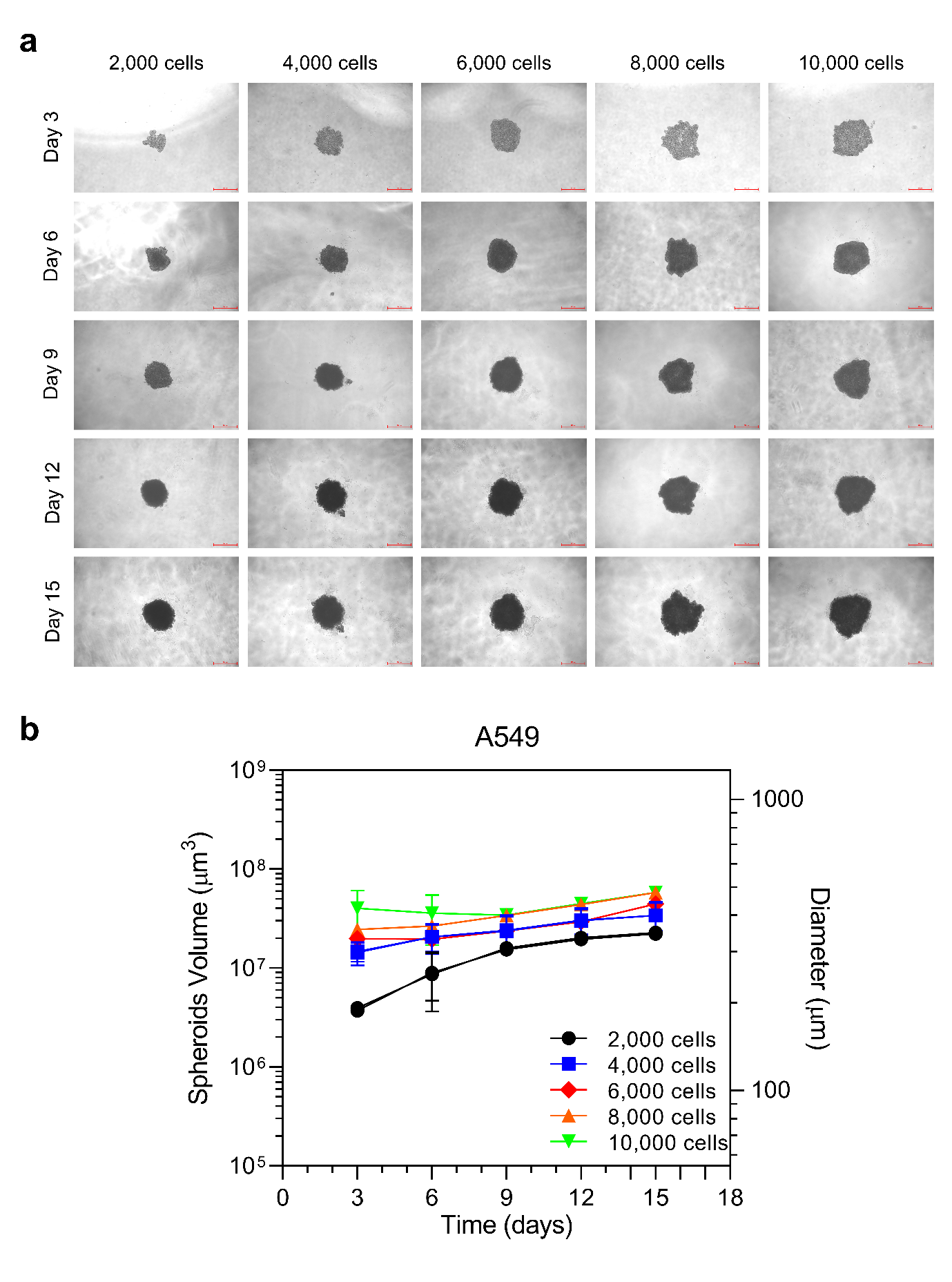
**

**Figure S4.** A549 spheroids growth. a) Spheroids of A549 lung cancer cells upon seeding at 2000, 4000, 6000, 8000 and 10000 cells/well and images were photographed using light microscope at day 3, 6, 9, 12 and 15. Scale bar = 500 µm. b) Spheroids volume for A549 lung cancer cells. Mean ± SD of eighteen spheroids from three independent experiments.
